# Design, Synthesis, and Application in OFET of a Quinoxaline-Based D-A Conjugated Polymer

**DOI:** 10.3389/fchem.2022.934203

**Published:** 2022-06-16

**Authors:** Zhicheng Dai, Daohai Zhang, Haichang Zhang

**Affiliations:** ^1^ Key Laboratory of Rubber-Plastics of Ministry of Education/Shandong Province (QUST), School of Polymer Science and Engineering, Qingdao University of Science and Technology, Qingdao, China; ^2^ School of Chemical Engineering of Guizhou Minzu University, Guiyang, China

**Keywords:** organic field-effect transistor, conjugated polymers, quinoxaline, donor, acceptor

## Abstract

A novel alternating donor–acceptor polymer PQ1 is designed and synthesized by palladium-catalyzed Stille coupling between quinoxaline as an electron-deficient unit and indacenodithiophene (IDT) as electron-rich groups. Polymer PQ1 presents not only a strong intramolecular charge transfer effect, which is beneficial for the charge transport within single molecules but also a narrow electrochemical band gap and a high highest occupied molecular orbital (HOMO) energy level. In addition, the optical absorption study indicates that the PQ1 film exhibits good aggregation, which is an advantage for the charge transport between neighboring molecules. As a consequence, PQ1 presents p-type semiconductor properties with a high hole mobility of up to 0.12 cm^2^ V^−1^ s^−1^. This study reveals the great potential of quinoxaline-type chromophores in constructing novel organic semiconductors.

## Introduction

Since the first organic field-effect transistor (OFET) was successfully prepared by Tsumura with polythiophene in 1987, organic semiconductors have received more and more attention from the academic society due to their multi-advantages and potential applications such as low cost, ease of fabrication, and compatibility with flexible substrates (Tsumura et al., 1986; Bogdanov and Mironov, 2021; Wang et al., 2020; Yoon et al., 2017; Zhang et al., 2018). In the past few years, OFETs have made significant achievements in charge carrier mobility, open-circuit voltage (V_oc_), and current on/off ratio. Regarding semiconductor materials, most used are the organic π-conjugated small molecules and polymers (Cao et al., 2012; Donaghey et al., 2013; Zhang et al., 2020). Compared to small molecules, polymers are more popular and widely used as semiconductor layers in high-performance OFETs, since they are often present not only in high intracharge transport mobility but also in tunable chemical structures (Kettle et al., 2011; Sasikumar et al., 2016; Bhanuprakash et al., 2016; Bi et al., 2018). According to the composition of the polymer backbone, semiconductor polymers are usually classified into three types: A-A type (acceptor–acceptor), D-D type (donor–donor), and D-A type (donor–acceptor). Among them, the D-A type is the most popular and potential in high-performance OFETs due to its convenient molecular orbital modification capability and good intramolecular charge transfer (ICT) effect (Tomasz et al., 2013; Deng et al., 2018a). A suitable donor structure can modulate the highest occupied molecular orbital (HOMO), while a suitable acceptor group might adjust the lowest unoccupied molecular orbital (LUMO). This results in semiconductor materials with an appropriate Frontier molecular orbital level, which aligns with the working function of the electrode, in turn forming an Ohmic contact with it, and facilitates charge injection from the electrode into the semiconductor layer.

Thiophene is a commonly used electron donor unit in π-conjugated polymers. Indacenodithiophene (IDT) belongs to a derivative of thiophene with conjugation system extension. With its excellent planar symmetric structure, it can effectively promote intermolecular charge transfer. Thus, the devices prepared by this structure usually have excellent charge carrier mobility (Cai et al., 2014; Zhai and Zhou, 2016). Recent research has reversed that IDT has become a widely used feeder structure for photovoltaic materials, especially in organic solar cells (OPV), which can provide an energy conversion efficiency of over 9.21% (Andrew et al., 2020). In addition, it is also used as donor units to construct D-A type semiconductors with good hole transport mobility. Apart from the donor unit, a suitable acceptor group also plays a crucial role in designing a high-performance semiconductor (Kettle et al., 2011). The derivatives of quinoxaline, an electron-deficient chromophore, play an important role as a good planar and rigid conjugated structure in organic light-emitting diodes (OLEDs), dyes, or as ligands in light-emitting materials, but are rarely used in the construction of semiconductors in OFETs (Liu et al., 2017; Park et al., 2019; Hee et al., 2020; Sagita, et al., 2021).

In this work, a novel polymer PQ1 is designed and synthesized by the Stille coupling reaction between IDT and thiophene-substituted quinoxaline (Supplementary Scheme S1) (Li et al., 2012; Deng et al., 2018b). In order to improve the solubility of the polymer, multiple large sized alky chains are introduced into the polymer. The optical and electrochemical properties of the polymer are investigated. In addition, the OFETs are constructed using PQ1 as the semiconductor layer, which presents a hole mobility of around 0.1 cm^2^ V^−1^ s^−1^.

## Results and Discussion

### Computational Study

**FIGURE 1:**
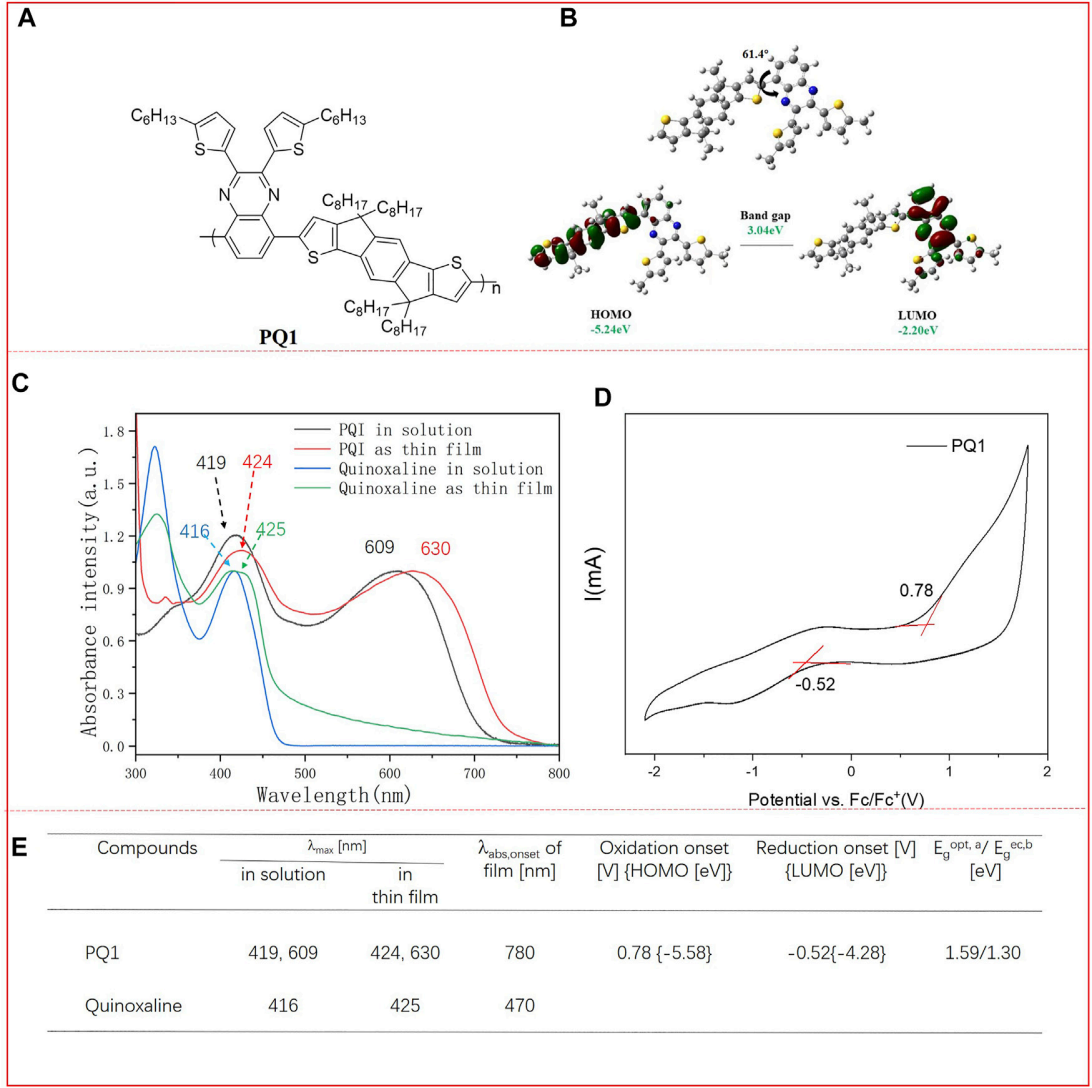
**(A)** Chemical structures of PQ1; **(B)** computational calculations of the simplified single repeat unit of the polymer obtained at the B3LYP/6-31G* level; **(C)** UV/vis absorption spectra of the monomer quinoxanline and polymer PQ1 in dichloromethane solution and the polymer thin film; **(D)** cyclic voltammograms of the polymer PQ1 as thin films deposited on ITO electrolyte: 0.1 M TBAPF_6_/acetonitrile. Potential calculated versus ferrocene. Scan rate: 100 mV s ^−1^; T = 25°C. **(E)** Optical and electrochemical properties of the monomer and polymer PQ1. ^a^E_opt_ (optical bandgap) was measured at the onset of absorption in the film (E_opt_ = 1,240/*?*_abs. onset_ eV). ^b^E_g_^ec^ electrochemical bandgap. HOMO-LUMO gap was calculated according to the following equations: E_LUMO_ = E_onset (red)_ + 4.8 eV and E_HOMO_ = E_onset (ox)_ + 4.8 eV. E_onset (ox)_ and E_onset (red)_ are onset potentials for the oxidation and reduction processes *vs* Fc/Fc^+^ couple, respectively.

In order to investigate the Frontier molecular orbital features, backbone configuration, and the HOMO/LUMO energy levels of the two polymers, computational calculations were conducted by density-functional theory (DFT) at the B3LYP/6-31(d,p) level using the model compound containing a single repeating unit with a methyl group instead of an alky chain. As shown in Figure 1A, there is a 61.4 twisting angle between the donor units of IDT and the acceptor unit of quinoxaline. The electronic cloud distributions of LUMO are mainly located at the IDT part, while the LUMO electrons are localized on the quinoxaline groups. This indicates that once the polymer is excited, an electron transfer from the IDT part to the quinoxaline groups could take place, which means that the polymer has a stronger ICT effect. A good ICT effect is beneficial for the charge transport within the individual molecules. The calculated HOMO/LUMO level of PQ1 is -5.24 and -2.20 eV, resulting in a band gap of 3.04 eV.

### Optical Properties

In order to evaluate the optical properties, UV/vis spectroscopy of the PQ1 polymer is performed in a chloroform solution and thin-film state. The corresponding optical data are summarized in Supplementary Table S1. In Figure 1A, the UV/Vis absorption spectra of quinoxaline and PQI in dichloromethane and in the thin-film state are shown. The polymer PQ1 presents dark blue color in both the solution and thin-film state. The absorption spectrum of the quinoxaline in dichloromethane exhibited a strong absorption maximum (*λ*_abs.max_) at 416 nm with an extinction coefficient of 3.2×10^4^ L mol^−1^ cm^−1^, which was only blue-shifted around 9 nm compared to its thin-film state (*λ*_abs.max_ = 425 nm). After polymerization, absorption of polymer PQ1 exhibited a large shift (around 193 nm) to a longer wavelength in the solutions compared to the monomer (from 416 to 609 nm). The red-shift could be due to the π-conjugation extension and the donor–acceptor interaction within the polymer backbones, which inevitably caused the intramolecular charge transfer (ICT) process to generate more delocalized intramolecular π-orbitals and thus increased the efficient conjugation lengths^[23]^ Regarding the polymer film, a 21-nm red-shifted absorption was observed compared to their solution counterparts. In addition, in the long-wavelength absorption range, the bathochromic shift appeared to be stronger (around 50 nm). These observations could be due to the aggregation, the π–π interaction, and the long-range ordered packing. From the onset of absorption, the optical band gap was calculated to be 1.59 eV.

### Electrochemical Properties

The electrochemical properties of the polymer were investigated by cyclic voltammetry. The experimental details are described in the Supporting Information. Based on the onset reduction and oxidation potentials, the LUMO/HOMO energy levels of the monomer and polymers are estimated (Guo et al., 2014). As shown in Figure 1D, PQ1 presents quasi-reversible cathodic and anodic waves. The onset oxidation occurred at 0.78 V and reduction at -0.52 V, based on which the HOMO and LUMO energy levels were calculated to be -5.58 eV and -4.28 eV, respectively. The HOMO energy level of PQ1 is lower than the oxidation threshold of air, i.e., −5.27 eV, indicating the good stability of the materials in air. According to the HOMO/LUMO energy levels, the electrochemical band gap was calculated to be 1.3 eV. This value is smaller than the computation results, which is ascribed to the fact that the computation result is obtained using the single repeat unit, while the electrochemical band gap is obtained using the whole polymer. In addition, the electrochemical band gap is also 0.2 eV smaller than the optical band gap.

### OFET Device

**FIGURE 2:**
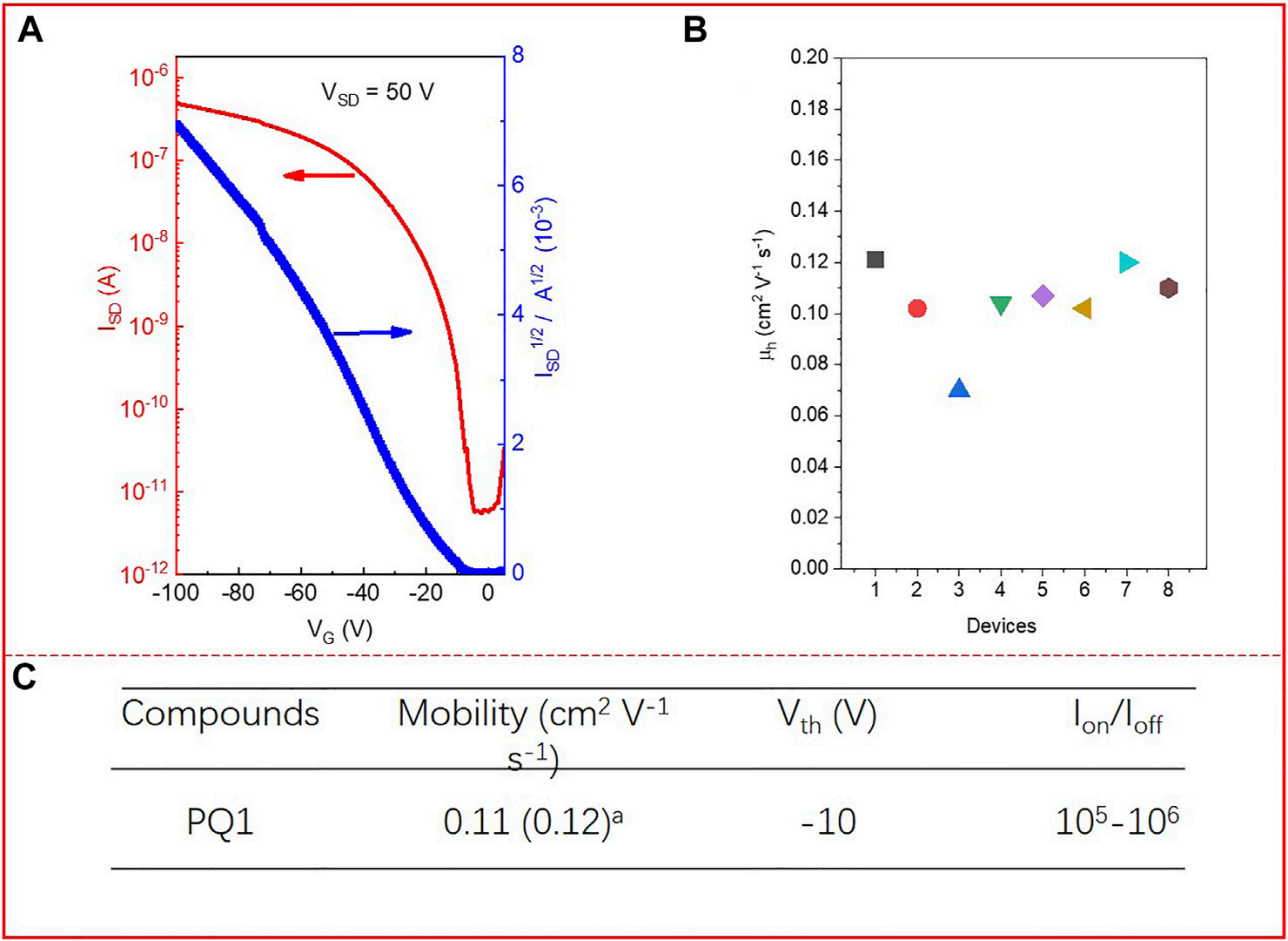
**(A)** Characteristics of the OFET devices of PQ1; **(B)** hole transport mobility obtained eight different devices. **(C)** Hole mobilities (µ_h_), threshold voltage (V_Th_), and on/off ratios (I_on_/I_off_) of the PQ1-based FET device. The mobility was provided in average (highest)^a^ form and the performance is based on eight different FETs. Mobility extracted by fitting the linear part of the plot of I_DS_^1/2^ versus V_G_ using the equation I_DS_ = C_i_µ(V_G_-V_Th_)^2^W/2L. ^a^ Hole mobility.

The charge transport properties of the PQ1 polymer are evaluated by fabricating the OFET devices of the materials with bottom-gate and bottom-contact (BGBC) configuration on a silicon wafer using a layer of 300 nm SiO_2_ as the dielectric material. The devices with PQ1 IS were fabricated by direct spin-casting of the polymer solution in toluene onto the OTS-treated silicon wafer with prepatterned gold source and drain electrodes, and measured under vacuum conditions. The devices were measured after thermal annealing at 100°C for 30 min in an argon-filled glove box to remove the remaining solvent residues. The detailed fabrication and testing procedures are described in the Supporting Information. As shown in Figure 2, the PQ1 polymer presents a p-type charge transport behavior with a hole mobility (μ_h_) estimated to be 0.11 cm^2^ V^−1^ s^−1^ (average) for eight different devices. From the eight devices, the highest μ_h_ is 0.12 cm^2^ V^−1^ s^−1^. The high hole mobility could be ascribed to the strong ICT effect and the good aggregation (Du et al., 2018; Muhammad et al., 2020).

## Conclusion

In conclusion, a D-A type polymer PQ1 is successfully designed and synthesized between quinoxaline as the electron-deficient unit and IDT as the electron-rich unit by the Stille coupling reaction. UV-vis spectra showed that after polymerization, there is a large red-shift from the monomer to the polymer, which indicates the strong ICT effect for D-A polymer PQ1. In addition, there is a 21-nm bathochromic shift from the solution to thin film for PQ1, which indicates that the PQ1 film presents good aggregation. The electrochemical study indicates that PQ1 exhibits a low band gap with a HOMO energy level of −5.48 eV, which is lower than the oxidation threshold of air, i.e., −5.27 eV, indicating the good stability of PQ1 in air. Using PQ1 as the semiconductor layer to construct OFET presents p-type behavior with a hole mobility of up to 0.12 cm^2^ V^−1^ s^−1^. This study demonstrated the great potential of quinoxaline-type chromophores in constructing novel organic semiconductors, and thus, further modification of quinoxaline structures is currently underway in our laboratory.

## Data Availability

The datasets presented in this study can be found in online repositories. The names of the repository/repositories and accession number(s) can be found in the article/Supplementary Material.
