# Supplementary material for: Design, Synthesis, and Application in OFET of a Quinoxaline-Based D-A Conjugated Polymer
Source: Front Chem. 2022 Jun 16;10:934203. doi: 10.3389/fchem.2022.934203 (PMC9244549; doi:10.3389/fchem.2022.934203)
Supplement: Supplementary file 1 [file DataSheet1.docx]

**Supporting Information**

**Design, Synthesis and Application in OFET of a Quinoxaline-based D-A Conjugated Polymer**

**Daohai Zhang,^1a*^ Zhicheng Dai,^1b^ Haichang Zhang*^b^**

^a^ School of Chemical Engineering of Guizhou Minzu University, Guizhou,Guiyang 550025, China,

^b^ Key Laboratory of Rubber-Plastics of Ministry of Education/Shandong Province (QUST), School of Polymer Science & Engineering, Qingdao University of Science & Technology, 53-Zhengzhou Road, Qingdao, 266042, PR China

Corresponding author: Daohai Zhang: [zhangdaohai6235@163.com](mailto:zhangdaohai6235@163.com); Haichang Zhang: [haichangzhang@hotmail.com](mailto:haichangzhang@hotmail.com)

**Contents**

**1. Experimental Procedures**

1.1 Materials

1.2 Synthesis

1.2.1 Synthesis of compound 1

Scheme S1. Synthetic route to PQ1.

1.2.2 Synthesis of compound 2

1.2.3 Synthesis of compound 3

1.2.4 Synthesis of compound PQ1

1.3 Characterization for PQ1

1.3.1 Structure characterization

1.3.2 Electrochemical properties measurements

1.3.3 UV/vis absorption measurements

1.3.4 Thin film structures characterization

1.3.5 OFET devices fabrication

**2. Results**

2.1 Figures

Figure S1. ^1^H NMR and ^13^C NMR spectra of compound 1.

Figure S2. ^1^H NMR and ^13^C NMR spectra of compound 2.

Figure S3. ^1^H NMR and ^13^C NMR spectra of compound 3.

Figure S4. ^1^H NMR and ^13^C NMR spectra of PQ1.

**3. References**

# **Experimental Section**

## *1.1 Materials*

All reagents were purchased from commercial sources and used without further purification unless otherwise noted. 4,7-Dibromo-2,1,3-benzothiadiazole, 2-Hexylthiophene, Sodium borohydride, Lithium bromide, Copper(I)bromide, K_2_CO_3_, Pd(PPh_3_)_4_, , and the solvents were obtained from Energy Chemical.

## *1.2 Synthesis*

Scheme S1. Synthetic route to PQ1

## *1.2.1 Synthesis of compound 1*

In a 100 ml one-neck flask, dissolve 4,7-Dibromo-2,1,3-benzothiadiazole (1.2g, 4mmol) in 40 mL of ethanol. After cooling to 0°C, sodium borohydride (0.37g,10mmol) was added. After stirring for 1 hour, the reaction was carried out at room temperature for 12 hours. Subsequently, it was extracted with dichloromethane and deionized water twice and dried over anhydrous MgSO_4_. The crude product was purified by column chromatography by gradient elution method (silica gel, petroleum ether: dichloromethane = 1:1, dichloromethane: methanol =25:2 v:v) to afford compound 1 (0.46 g, yield: 43 %) as a white solid.^[1] 1^H NMR (500 MHz, CDCl_3_-d_1_) δ ppm: 6.84 (s,2H), 3.99 (s, 24). ^13^C NMR (500 MHz, CDCl_3_ d_1_) δ ppm: 133.73, 122.70, 109.01.

## *1.2.2 Synthesis of compound 2*

In a N_2_-protected 100 mL single-necked flask, 2-Hexylthiophene (2.52 g, 15 mmol) was dissolved in 20 mL of THF at 0 °C, to which a hexane solution of butyllithium (6.52 mL, 2.3 M, 15 mmol) was subsequently added dropwise. Anhydrous LiBr (2.76 g, 32 mmol) was dissolved in THF (40 mL) in another 250 mL single-necked flask protected by N2, to which anhydrous CuBr (2.16 g, 15 mmol) dissolved in THF (40 mL) was added, cooled to room temperature and stirred well. Subsequently, the THF solution of 2-Hexylthiophene and butyllithium was poured into it and oxalyl chloride (0.86 g, 6.8 mmol) was added dropwise after stirring for 30 min. After stirring at 0 °C for 1 h, 100 mL of aqueous solution of saturated ammonium chloride was added to the reaction system. Subsequently, it was extracted with dichloromethane and deionized water twice and dried over anhydrous MgSO_4_. The crude product was purified by column chromatography (silica gel, petroleum ether: dichloromethane = 3:1 v:v) to afford compound 2 (1.25 yield: 57.6%) as a golden yellow solid. ^[1] 1^H NMR (500 MHz, CDCl_3_-d_1_) δ ppm: 7.86-7.85 (d, 4H), 6.88-6.87 (d, 4H), 2.89-2.86 (t, 4H), 1.74-1.68 (m, 4H), 1.40-1.34 (m, 4H), 1.32-1.28 (m, 8H), 0.90-0.87 (t, 6H). ^13^C NMR (500 MHz, CDCl_3_ d_1_) δ ppm: 182.63, 160.07, 137.77, 136.51, 126.42, 31.46, 31.24, 30.83, 28.69, 22.52, 14.04

## *1.2.3 Synthesis of compound 3*

Compound 1 (1.33 g, 5 mmol) and compound 2 (1.95 g, 5 mmol) were dissolved in 70 mL of acetic acid in a N_2_-protected 250 mL single-neck flask. After stirring for 5 hours, it was filtered and washed with ethanol. Compound 3 (1.6 g, yield: 63 %) was obtained as a yellow-green solid after drying. ^[1] 1^H NMR (500 MHz, CDCl_3_-d_1_) δ ppm: 7.77 (s,2H), 7.41-7.40 (d, 2H), 6.71 (d, 2H), 2.88-2.85 (t, 4H), 1.77-1.73 (m, 4H), 1.42-1.38 (t, 4H), 1.35-1.32 (m, 8H), 0.92-0.89 (m, 6H). ^13^C NMR (500 MHz, CDCl_3_ d_1_) δ ppm: 152.08, 147.21, 138.47, 138.30, 132.57, 130.35, 124.96, 122.86, 31.55, 31.49, 30.46, 28.86, 22.58, 14.09.

## *1.2.4 Synthesis of PQ1*

In a 50 ml round bottom flask, 104.9 mg of IDT (0.1 mmol) and 65.1 mg of quinoxaline (0.1 mmol) were dissolved in 15 ml of toluene). The mixture was degassed for 10 min under N_2_ protection using a vacuum oil pump. 3.3 mg of Pd(PPh_3_)_4_ (0.028 mmol) was added to the mixture rapidly under N_2_ protection. Subsequently, the mixture was degassed at room temperature for 5 min. The reaction temperature was increased to 100 °C until solvent reflux. After 24 h, the temperature was lowered and the dark blue solution was added dropwise to the rapidly stirred methanol. After filtration 97.5 mg of blue-black solid PQ1 was obtained (yield: 83%). ^1^H NMR (500 MHz, CDCl_3_-d_1_) δ ppm: 8.07-8.06 (s,2H), 7.87-7.84 (d, 2H), 7.80-7.78 (d, 2H), 7.33-7.31 (d, 2H), 7.05 (s, 2H), 2.84-2.80 (t, 4H), 1.96-1.78 (m, 12H), 1.30-1.26 (m, 60H), 0.91-0.85 (m, 18H).

## *1.3 Characterization for the polymer*

## *1.3.1 Structure characterization*

NMR spectra were obtained using a Mercury 500 spectrometer. Elemental analysis was performed on a Carlo Erba 1106 Elemental Analyzer.

## *1.3.2 Electrochemical properties measurements*

Cyclic voltammetry (CV) measurements were performed with a BAS 100 W Bioanalytical Systems, using a glass carbon disk (Φ = 3 mm) as the working electrode, a platinum wire as the auxiliary electrode with a porous ceramic wick, and Ag/Ag^+^ as the reference electrode, standardized for the redox couple ferricinium/ferrocene. All solutions were purged with a nitrogen stream for 10 min before measurement. The procedure was performed at room temperature and a nitrogen atmosphere was maintained during the measurements.

## *1.3.3 UV/vis absorption measurements*

UV/vis absorption spectra were recorded using a dual-beam grating Agilent Cary 5000 absorption spectrometer. The thin film UV/Vis absorption spectra of these two molecules were measured by using spin-coated thin film (8mg/mL molecules in chloroform spin-coated on quartz glass substrate, rotation speed: 1500 rpm).

## *1.3.4 Thin film structures characterization*

Thin film X-ray diffraction (XRD) experiments working at 3 KW were performed on a Powder X-ray Diffractometry (INCA Energy, Oxford Instruments). The films were prepared by spin-coasting of polymer’ solution (8 mg / ml in chloroform).

## *1.3.5 OFET devices fabrication*

Bottom gate, top contact thin-film field effect transistors configuration was used to evaluate the polymer semiconductors. Highly doped Si wafer with 300 nm thermal oxide (Silicon Quest International) was used as the substrate, where the doped silicon layer was used as back gate (G) and the oxide as the dielectric materials (*C_i_*, capacitance of 10 nF·cm^–2^). After cleaning the substrate with Piranha (H_2_SO_4_/H_2_O_2_ = 3/1), DI water and acetone, plasma clean (10 min, PDC-001), the substrate was put in OTS solution (5 % in toluene) at room temperature for over nigh in Ar-filled glove box. Subsequently the source (S) and drain (D) electrode pairs were electron-beam evaporated on the surface of the silicon wafer by using Au target (with a thickness of 50 nm) through a mask. The semiconductor layer was deposited though spin-coated by the polymer’ solution (8 mg/mL in chloroform). The polymer transistor devices were further treatment by thermal annealing at 50 ^o^C for 1 h in order to make sure all the chloroform evaporation. The TFT device has a channel length (*L*) of 50 µm with a channel width (*W*) of 1 mm. The field effect mobility was calculated using *I*_SD_ = (*W*/2*L*)·*µC*_i_ (*V*_G_ – *V*_T_)^2^, where *L* and *W* are the channel length and width; *C_i_* is the capacitance of gate oxide, *V_SD_* is voltage between source and drain electrodes; *I_SD_* is the current between source and drain electrodes; *V_G_* is the gate voltage.

# **2. Results**

##
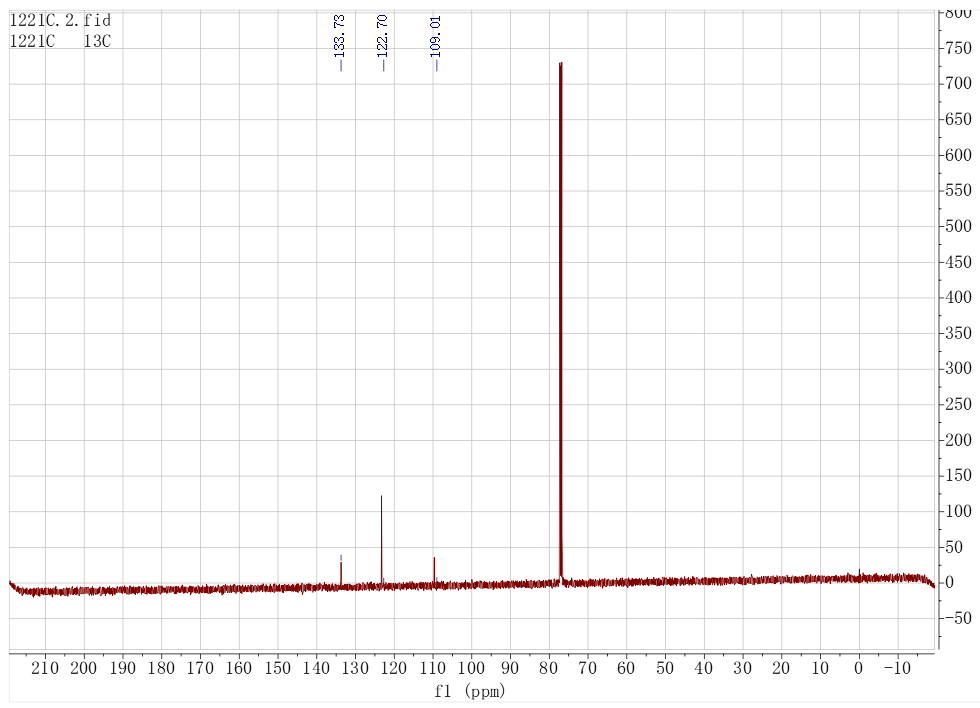

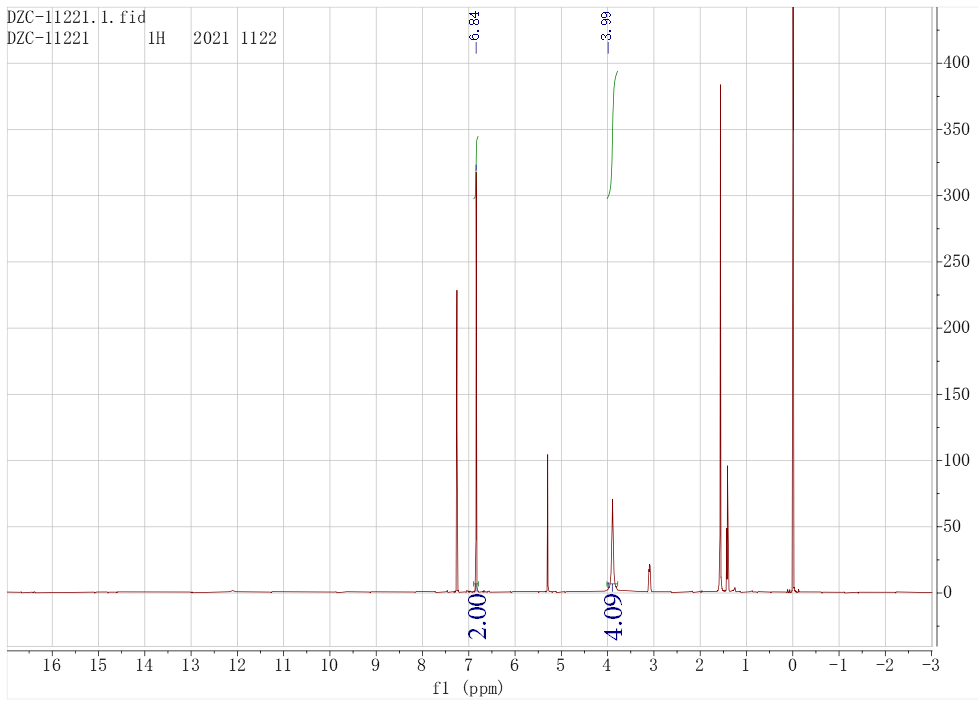
*2.1 NMR of compound 1*

**Figure S1.** ^1^H NMR and ^13^C NMR spectra of compound 1

##
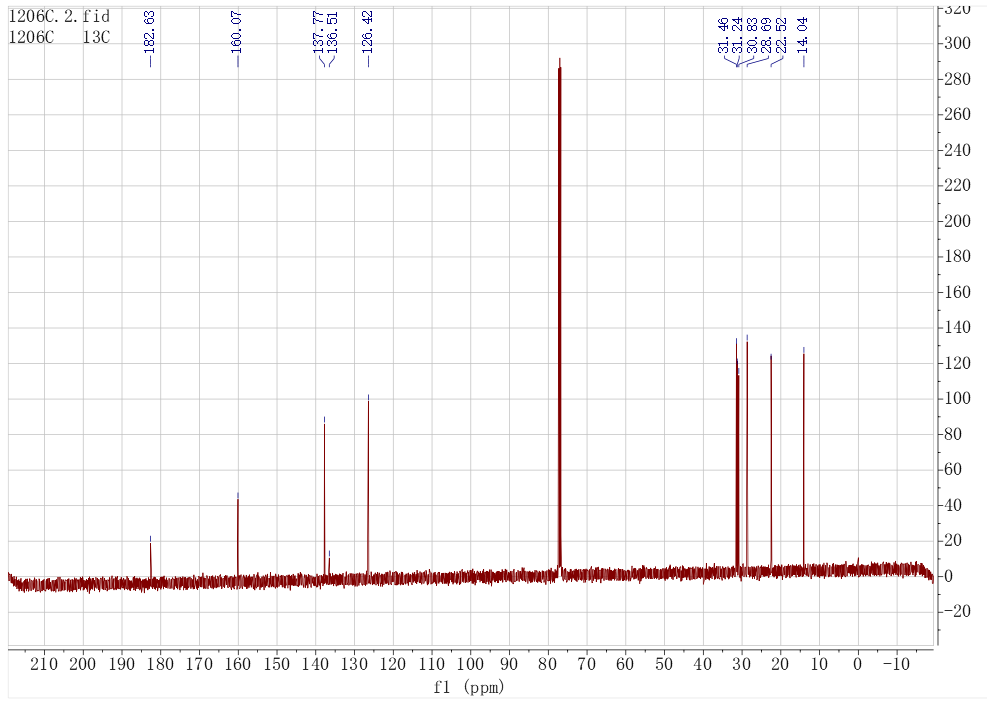

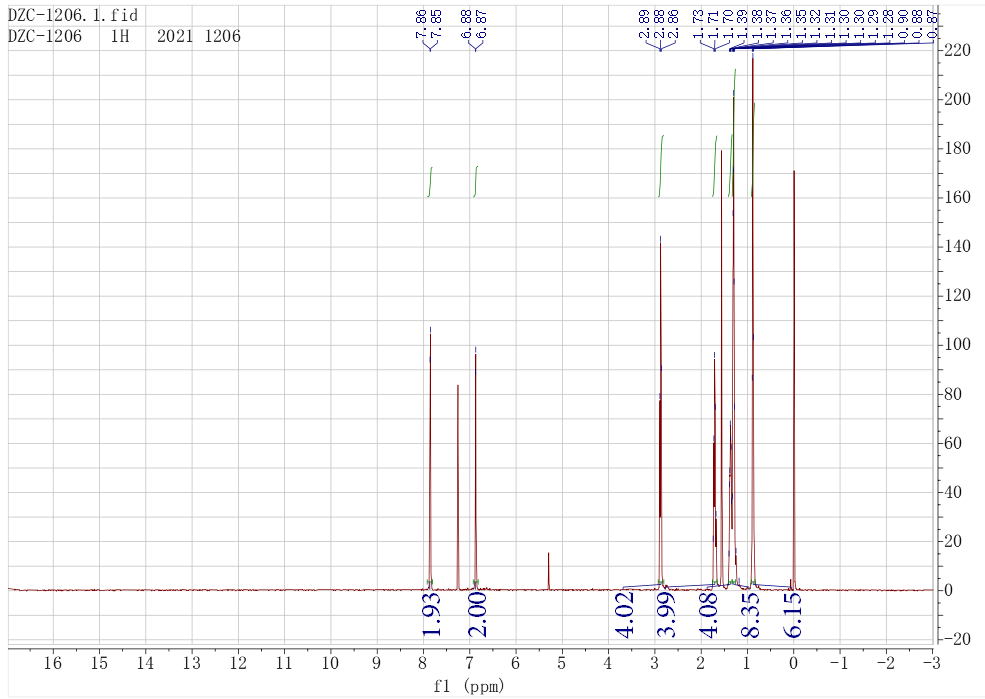
*NMR of compound 2*

**Figure S2.** ^1^H NMR and ^13^C NMR spectra of compound 2

##
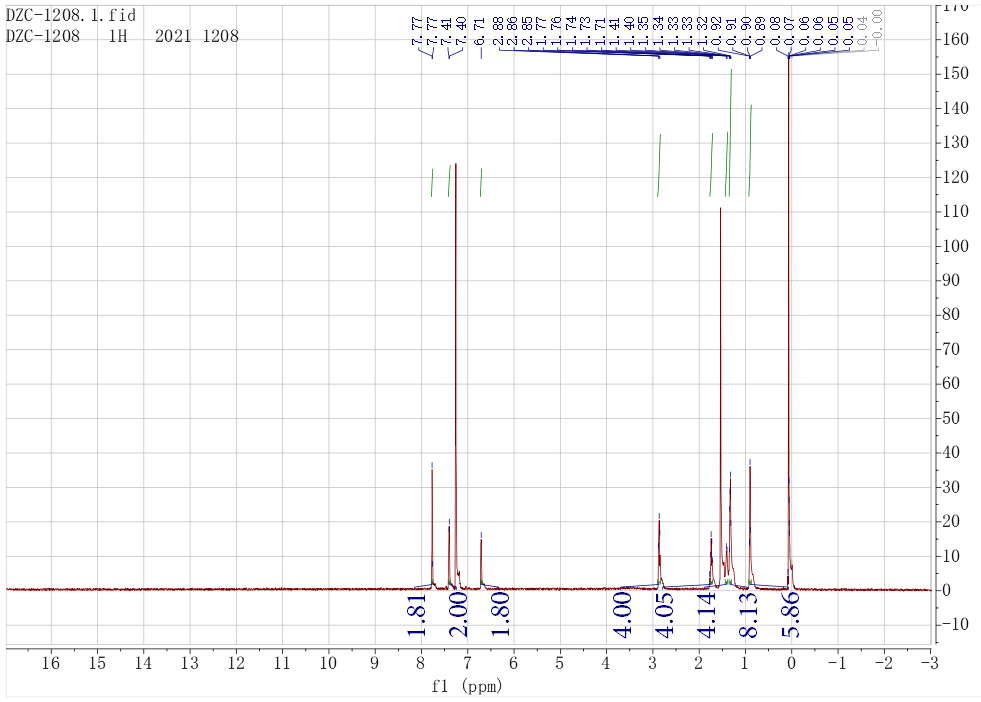

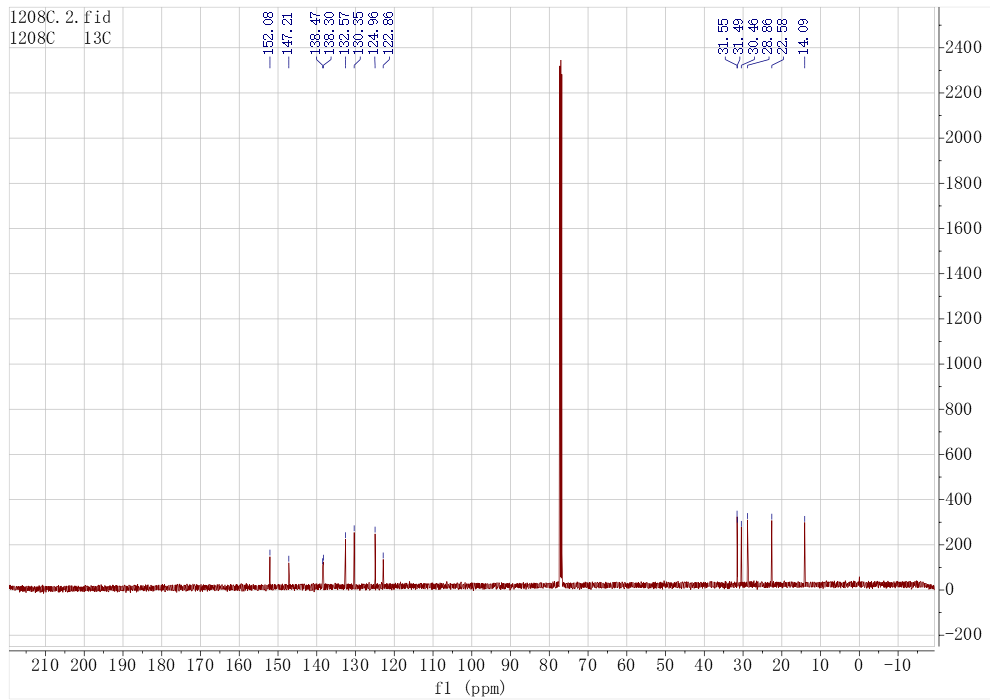
*2.3 NMR of compound 3*

**Figure S3.** ^1^H NMR and ^13^C NMR spectra of compound 3

## *
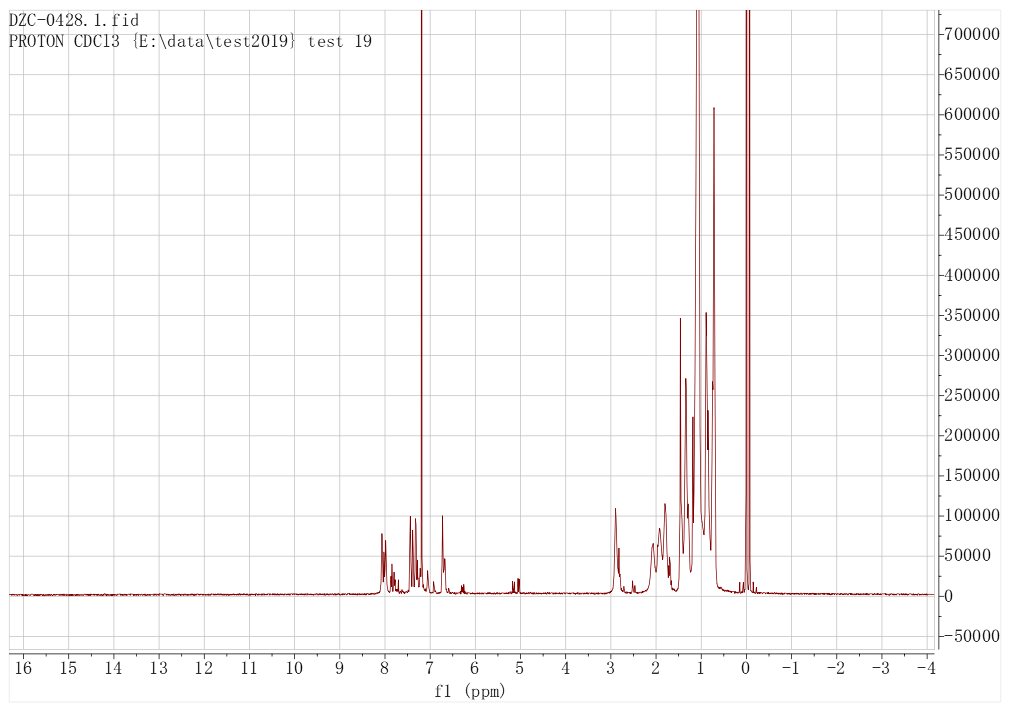
2.4 NMR of PQ1*

**Figure S4.** ^1^H NMR and ^13^C NMR spectra of PQ1

# **3. References**

[1] Li S, He Z, Yu J, Chen S, Zhong A, Wu H, Zhong C, Qin J, Li Z. 2,3-bis(5-Hexylthiophen-2-yl)-6,7-bis(octyloxy)-5,8-di(thiophen-2-yl) quinoxaline: A good construction block with adjustable role in the donor-π-acceptor system for bulk-heterojunction solar cells. J Polym Sci Pol Chem 2012;50:2819-28
